# Family Dysfunction Differentially Affects Alcohol and Methamphetamine Dependence: A View from the Addiction Severity Index in Japan

**DOI:** 10.3390/ijerph8103922

**Published:** 2011-10-11

**Authors:** Nagisa Sugaya, Ayako Haraguchi, Yasukazu Ogai, Eiichi Senoo, Susumu Higuchi, Mitsuru Umeno, Yuzo Aikawa, Kazutaka Ikeda

**Affiliations:** 1 Tokyo Metropolitan Institute of Medical Science, 2-1-6 Kamikitazawa, Setagaya-ku, Tokyo 156-8506, Japan; E-Mails: sugaya-ng@igakuken.or.jp (N.S.); haraguchi-ay@igakuken.or.jp (A.H.); ogai.ys@md.tsukuba.ac.jp (Y.O.); senoo-ei@igakuken.or.jp (E.S.); 2 University of Tsukuba, 1-1-1 Tennodai, Tsukuba, Ibaraki 305-8577, Japan; 3 Ibaraki Prefectural Medical Center of Psychiatry, 654 Asahi-cho, Kasama-shi, Ibaraki 309-1717, Japan; 4 National Hospital Organization Kurihama Alcoholism Center, 5-3-1, Nobi, Yokosuka, Kanagawa 246-0841, Japan; E-Mail: h-susumu@db3.so-net.ne.jp; 5 Tokyo Metropolitan Matsuzawa Hospital, 2-1-1 Kamikitazawa, Setagaya-ku, Tokyo, 156-0057, Japan; E-Mails: ve5m-umn@asahi-net.or.jp (M.U.); yuzoaikawa@yahoo.co.jp (Y.A.)

**Keywords:** alcohol dependence, methamphetamine dependence, Addiction Severity Index, family relationship

## Abstract

We investigated the differential influence of family dysfunction on alcohol and methamphetamine dependence in Japan using the Addiction Severity Index (ASI), a useful instrument that multilaterally measures the severity of substance dependence. The participants in this study were 321 male patients with alcohol dependence and 68 male patients with methamphetamine dependence. We conducted semi-structured interviews with each patient using the ASI, which is designed to assess problem severity in seven functional domains: Medical, Employment/Support, Alcohol use, Drug use, Legal, Family/Social relationships, and Psychiatric. In patients with alcohol dependence, bad relationships with parents, brothers and sisters, and friends in their lives were related to current severe psychiatric problems. Bad relationships with brothers and sisters and partners in their lives were related to current severe employment/support problems, and bad relationships with partners in their lives were related to current severe family/social problems. The current severity of psychiatric problems was related to the current severity of drug use and family/social problems in patients with alcohol dependence. Patients with methamphetamine dependence had difficulty developing good relationships with their father. Furthermore, the current severity of psychiatric problems was related to the current severity of medical, employment/support, and family/social problems in patients with methamphetamine dependence. The results of this study suggest that family dysfunction differentially affects alcohol and methamphetamine dependence. Additionally, family relationships may be particularly related to psychiatric problems in these patients, although the ASI was developed to independently evaluate each of seven problem areas.

## 1. Introduction

In 2003, approximately 800,000 adults of the Japanese general population of 120 million could be classified with alcohol dependence, making this group one of the largest among the various mental disorders [1]. Additionally, stimulant dependence is a serious problem not only for patients, but also for Japanese society [2]. For example, approximately 25% of convicted prisoners committed offenses under the Stimulant Control Law [3].

Previous studies have suggested that social support is an important factor for improving the symptoms of substance dependence. Coping and social support are related to substance use behavior and treatment outcomes in adolescents [4,5]. Social support also plays an important role in relapse avoidance efforts for individuals who undergo substance use treatment. Social support is a “social fund” from which individuals draw assistance when confronting stressors [6].

On the other hand, bad relationships may be an aggravating factor. Previous studies have reported an association between familial relationships and substance dependence. Multidimensional Family Therapy is uniquely suited to address adolescent substance abuse and related disorders, given its comprehensive interventions that systematically target the multiple interacting risk factors that underlie many of the developmental disruptions of adolescence [7]. A previous study of alcohol dependence suggested that among the many biological, morphological, and social markers of increased maturation, visible signs of maturity are important triggers of alcohol use and alcohol use disorders, especially when they occur early and in young people with conduct problems, deviant peers, problem families, and inadequate parental supervision [8]. Another study of drug dependence reported that drug use prevention should not simply focus on reducing drug availability, but also help young people develop good family/peer relationships and find healthy ways to enjoy themselves [9].

The Addiction Severity Index (ASI) is a semi-structured clinical research interview widely used in substance abuse treatment settings in the United States and many other countries. This instrument was designed to assess problem severity in seven functional domains: Medical, Employment/Support, Alcohol use, Drug use, Legal, Family/Social relationships, and Psychiatric [10]. Therefore, family relationships are an important factor in assessing the severity of substance dependence using the ASI.

A comparison of the characteristics of family relationships and the association between family relationships and various problems related to substance dependence in patients with alcohol and drug dependence using the ASI may be useful for establishing personalized programs for individuals with substance dependence. However, no study of which we are aware has compared the differences in the association between family dysfunction and problems related to substance dependence between alcohol and drug dependence. Moreover, the ratio of individuals who use methamphetamine is the highest in individuals with drug dependence in Japanese hospitals, suggesting that it may be meaningful to focus on the characteristics of individuals with methamphetamine dependence. Therefore, we investigated the differences in the influence of family dysfunction on alcohol dependence and methamphetamine dependence in Japanese patients using the ASI as an exploratory survey. We hypothesized that family dysfunction in patients with alcohol and patients with methamphetamine dependence may be related to different aspects of problems related to substance dependence. The present exploratory study may provide future direction for more detailed investigations that lead to the development of more effective methods for finding appropriate psychological interventions for each patient.

## 2. Methods

### 2.1. Participants

We surveyed 370 patients with alcohol dependence and 83 patients with drug dependence. Valid data were obtained from 321 male patients with alcohol dependence (86.76%; mean age, 49.7 ± 11.0 years) and 80 male patients with drug dependence (96.39%; mean age, 32.9 ± 9.4 years). The participants with alcohol dependence were recruited from nine nationwide hospitals or recovery facilities for addiction treatment located in Japan: National Hospital Organization Kurihama Alcoholism Center, Kanagawa (*n* = 91), Wakamiya Hospital, Yamagata (*n* = 55), Komakino Hospital, Tokyo (*n* = 50), Mie Prefectural Mental Medical Center, Mie (*n* = 42), Asahiyama Hospital, Hokkaido (*n* = 26), Ishikawa Prefectural Takamatsu Hospital, Ishikawa (*n* = 17), National Hospital Organization Hizen Psychiatric Center, Saga (*n* = 14), Akagi-Kohgen Hospital, Gunma (*n* = 13), and Tohokukai Mental Hospital, Miyagi (*n* = 12). The participants with drug dependence were recruited from five nationwide hospitals or recovery facilities for addiction treatment in Japan: Tokyo Metropolitan Matsuzawa Hospital, Tokyo (*n* = 37), Self-Support Services (*i.e.*, a recovery facility run by a non-profit organization for addiction recovery), Tokyo (*n* = 16), National Center of Neurology and Psychiatry Musashi Hospital, Tokyo (*n* = 17), GAIA (i.e., a recovery facility run by a non-profit organization for addiction recovery), Okinawa (*n* = 8), and Fukko-kai Tarumi Hospital, Hyogo (*n* = 2).

### 2.2. Methods

The Japanese version of the ASI [11,12] was used in the present study. The ASI is a semi-structured clinical research interview designed to assess problem severity in seven functional domains: Medical status, Employment/Support status, Alcohol use, Drug use, Legal status, Family/Social relationships, and Psychiatric status [10]. The Medical status domain gathers basic information about medical history. It addresses information about lifetime hospitalizations, long-term medical problems, and recent physical ailments. The Employment/Support status domain gathers basic information about work experience and current sources of income. The Drug/Alcohol use domain gathers basic information about the patient's substance abuse history. It addresses information about current and lifetime substance abuse, the consequences of abuse, periods of abstinence, treatment episodes, and the financial burden of substance abuse. The Legal status domain gathers basic information about the patient's legal history. It addresses information about probation or parole, legal charges, convictions, incarcerations or detainments, and illegal activities. The Family/Social relationship domain assesses relationship problems with family members or friends. The Psychiatric status domain is used not to diagnose psychiatric disorders but to assess the experience of various psychiatric symptoms other than those associated with the effects of alcohol or drugs.

Acceptable reliability and validity of the ASI were confirmed in patients with drug [11] and alcohol dependence [12]. The ASI provides a composite score (CS). The CS in each problem area is a mathematically calculated score mainly based on patient responses to sets of items that ask the patient to report behaviors during the 30 days prior to the interview. The CS is calculated using a weighted formula designed to provide an equal contribution from each item and varies from 0 to 1, with a higher score indicating greater problem severity. Additionally, we analyzed the items of the ASI related to education years, employment status, marital status, cohabitation, years of current cohabitation, experience of abuse, family history of substance dependence or psychiatric disorders, and family relationships in their life.

### 2.3. Procedure

The recruitment criteria were the following: at least 18 years of age, a history of substance addiction problems diagnosed as alcohol dependence or drug dependence based on the criteria of the *Diagnostic and Statistical Manual of Mental Disorders*, 4th edition (DSM-IV), and the ability to understand Japanese.

The inpatients with alcohol dependence were provided an average 80-day treatment program (e.g., group meetings, alcohol education, family treatment programs, psychotherapy, and so on) after detoxification. After recovery from serious physical and mental instability (nearly 1 month after hospitalization), informed consent was obtained from the subjects, excluding the patients who had serious cognitive impairment and psychiatric problems.

The participants with drug dependence were inpatients or outpatients at a Japanese mental hospital or recovery facility or non-patients who were recovering from stimulant abuse in a recovery facility. Considering the time required for an interview and the reliability of the responses, we excluded patients in a state of acute drug-induced psychosis.

The ASI was administered by psychiatrists and clinical psychologists who were experts in alcohol or drug dependence, carefully read the ASI manual [13], and learned the interview methods themselves. The average time required for administration of the questionnaire was 60 min. Inpatient subjects were requested to answer the questions during the 30 days prior to the start of inpatient treatment. The Institutional Review Board of each institution approved the study, and all of the participants provided written informed consent.

### 2.4. Statistical Analysis

Comparisons between groups with regard to age, number of convictions, and ASI CS were conducted using the *t*-test. Comparisons between groups with regard to the characteristics of education, employment, marital status, cohabitation, experience of abuse, and psychiatric symptoms were performed using the *x^2^* test and Fisher exact test (multiple comparisons were performed using residual analysis). The relationships between ASI CSs were analyzed using partial correlation analysis. The significance level was set at less than 0.05 or 0.01. Statistical analyses were performed with SPSS 18 (SPSS Inc., Chicago, IL).

## 3. Results

### 3.1. Participant Characteristics

Table 1 shows the substances that the participants with drug dependence in this study mainly used. Most of the patients with drug dependence (85.00%) used methamphetamine, and others used cannabis (6.25%), inhalants (3.75%), analgesics/hypnotics/tranquilizers (2.50%), antitussive drugs (1.25%), or hallucinogens (1.25%). We performed the subsequent statistical analysis only in individuals who mainly used methamphetamine as patients with methamphetamine dependence (*n* = 68).

Table 2 shows the characteristics of the participants. The mean age of the patients with alcohol dependence was significantly higher than that of patients with methamphetamine dependence (*t* = 12.31, *p* < 0.0001). Significant differences were found in educational background (*z* = 17.72, *p* = 0.003). Patients with alcohol dependence had a higher ratio of being a junior high school graduate (*p* < 0.05), and patients with drug dependence had a higher ratio of being a high school dropout (*p* < 0.05). Significant differences were found in employment status (*z* = 36.26, *p* < 0.0001). Patients with alcohol dependence had higher ratios of full-time employment (*p* < 0.05) and retirement (*p* < 0.05). Patients with methamphetamine dependence had higher ratios of part-time employment (*p* < 0.05) and unemployment (*p* < 0.05). A significant difference was found in marital status (*z* = 64.08, *p* < 0.0001). Patients with alcohol dependence had a higher ratio of being married (*p* < 0.05). Patients with methamphetamine dependence had a higher ratio of never being married (*p* < 0.05). Significant differences were found in cohabitation (*z* = 62.71, *p* < 0.0001). More patients with alcohol dependence lived with their family (*p* < 0.05). More patients with methamphetamine dependence lived with their parents (*p* < 0.05). Significant differences were found in years at their current residence (*z* = 12.24, *p* = 0.002). More patients with alcohol dependence had lived in their current residence for more than 10 years (*p* < 0.05). More patients with methamphetamine dependence had lived in their current residence for less than 10 years (*p* < 0.05). With regard to abuse, patients with methamphetamine dependence had a higher ratio of physical abuse experience (*z* = 8.48, *p* = 0.0007). With regard to psychiatric symptoms in the past month, patients with methamphetamine dependence had higher ratios of “hallucinations” (*z* = 17.11, *p* = 0.0003) and “trouble understanding, concentrating, or remembering” (*z* = 16.57, *p* = 0.0002). Patients with methamphetamine dependence had more convictions (*t* = 5.35, *p* < 0.0001).

### 3.2. Relationship between ASI Composite Scores

Table 3 shows the correlations between ASI CSs after controlling for age. For patients with alcohol dependence, the CS of psychiatric problems was significantly correlated with the CSs of drug use (*r* = 0.27, *p* < 0.0001) and family/social problems (*r* = 0.28, *p* < 0.0001). For patients with methamphetamine dependence, the CS of psychiatric problems was significantly correlated with the CSs of medical problems (*r* = 0.33, *p* = 0.008), employment/support problems (*r* = 0.40, *p* = 0.001), drug use (*r* = 0.40, *p* = 0.0009), and family/social problems (*r* = 0.43, *p* = 0.0004). The CS of family/social problems was significantly correlated with the CS of medical problems (*r* = 0.33, *p* = 0.007) and legal problems (*r* = 0.35, *p* = 0.004).

Table 4 shows the comparison of ratios of each psychiatric symptom in the past month between groups of high and low CSs of Family/Social relationship problems. These groups were divided on the basis of median CSs of Family/Social relationships. In patients with alcohol dependence, high CSs of Family/Social relationship problems was associated with higher ratios of serious depression (*z* = 10.98, *p* = 0.001), serious anxiety or tension (*z* = 6.17, *p* = 0.02), and serious thoughts of suicide (*z* = 6.81, *p* = 0.01) than the low CS group. In patients with methamphetamine dependence, no significant difference was found between groups of high and low CSs of Family/Social relationship problems in ratio of each psychiatric symptom.

### 3.3. Family History of Alcohol Dependence, Methamphetamine Dependence, and Psychiatric Disorders

Table 5 shows the family histories of alcohol dependence, methamphetamine dependence, and psychiatric disorders in the participants of the present study. Of the patients with alcohol dependence, 36.33% had fathers with alcohol-related problems, 20.87% had uncles (paternal) with alcohol-related problems, and 25.94% had brothers with alcohol-related problems, and these ratios were significantly higher compared with patients with methamphetamine dependence (father, *z* = 7.97, *p* = 0.005; uncle, *z* = 6.31, *p* = 0.009; brother, *z* = 8.81, *p* = 0.002). Of the patients with methamphetamine dependence, 9.52% had brothers with drug-related problems, and the ratio was significantly higher than that of patients with alcohol dependence (*z* = 22.90, *p* = 0.005).

### 3.4. Comparisons of Family Relationships between Patients with Alcohol Dependence and Patients with Methamphetamine Dependence

In the Family/Social relationship domain, patients answered “Yes,” “No,” or “Neither” about whether they had a close, long-lasting, personal relationship with family members, partners, or friends in their life. Participants who answered “Yes” were assigned to the “good relationships group,” and participants who answered “No” were assigned to the “bad relationships group.” In the comparison of experience of good relationships with family members (Table 6), patients with alcohol dependence had a significantly higher ratio of experience of good relationships with their father (*z* = 17.77, *p* < 0.0001).

### 3.5. Comparison of Severity of Addiction between Good and Bad Family Relationships

Tables 7 and 8 show the comparisons of ASI CSs between good and bad family relationships. Patients with alcohol dependence who experienced bad relationships with their brothers and sisters (*t* = 2.99, *p* = 0.003) and partners (*t* = 3.47, *p* = 0.0006) had a higher CS of employment/support problems. Patients who experienced bad relationships with their partners had a higher CS of family/social problems (*t* = 4.90, *p* < 0.0001). Patients who experienced bad relationships with their mothers (*t* = 2.73, *p* = 0.02), fathers (*t* = 2.84, *p* = 0.01), brothers and sisters (*t* = 2.82, *p* = 0.005), and friends (*t* = 2.99, *p* = 0.02) had a higher CS of psychiatric problems. In patients with methamphetamine dependence, no significant difference was found between good and bad family relationships in ASI CSs.

## 4. Discussion

With regard to the comparisons of family relationships between patients with alcohol dependence and patients with methamphetamine dependence, patients with methamphetamine dependence had difficulty developing good relationships with their father. With regard to the association between good relationships and the severity of substance dependence, in patients with alcohol dependence, bad relationships with parents, brothers and sisters, and friends were related to severe psychiatric problems. Bad relationships with brothers and sisters and partners were related to severe employment/support problems. Bad relationships with partners were related to severe family/social problems. In patients with methamphetamine dependence, no association was found between relationships and severity of substance dependence.

With regard to the associations between ASI CSs, psychiatric problems were related to drug use and family/social relationships in patients with alcohol dependence, and psychiatric problems were related to medical, employment/support, and family/social relationship problems in patients with methamphetamine dependence. In patients with alcohol dependence, relationships with various family members and friends were related to their mental condition, and bad relationships with their partners may be heavily involved in their difficult interpersonal relationships. Because problems with family/social relationships were related to psychiatric problems, bad relationships with their partners may be involved in psychiatric problems through their difficulties with interpersonal relationships. Additionally, the association between psychiatric problems and drug use in patients with alcohol dependence may be affected by the drugs prescribed for their psychiatric problems. Notably, some patients with alcohol dependence reported dependence on barbiturates or other analgesics/hypnotics/tranquilizers. Moreover, a deterioration of psychiatric problems may be involved in increased medical problems, employment/support problems, and drug use problems. These results suggest that although the ASI was developed to independently evaluate each of these seven problem areas [12], family relationships may be particularly related to psychiatric problems. Moreover, with regard to the associations between family/social relationships and specific symptoms, bad family/social relationships in alcohol dependence were related to the presence of serious depression, serious anxiety or tension, and serious thoughts of suicide, and bad family/social relationships in methamphetamine dependence were not related to the presence of specific psychiatric symptoms. Bad family/social relationships in patients with alcohol dependence and patients with methamphetamine dependence may be differentially related to psychiatric problems. Investigating the association between family relationships and psychiatric disorders may be useful, based on the relationship between family/social relationships and psychiatric status found in the present study.

The average age of the patients with alcohol dependence was higher than the average age of the patients with methamphetamine dependence, suggesting that having a long-term residence may be attributable to the higher average age of the patients with alcohol dependence. With regard to educational background, the higher ratio of junior high school graduation in patients with alcohol dependence may be attributable to the age group of patients with alcohol dependence, which contained many older patients. The higher ratio of being a high school dropout in patients with methamphetamine dependence may reflect their difficulty maintaining their relationships or completing their schoolwork on school days. With regard to employment status, the higher ratio of retirement in patients with alcohol dependence may be attributable to their higher average age, and the higher ratios of part-time employment and unemployment in patients with methamphetamine dependence may reflect their difficulty retaining a job. With regard to abuse experience, the higher ratio of being a victim of physical abuse in patients with methamphetamine dependence may make developing a trusting relationship with someone difficult. Consistent with this possibility, a previous study suggested that male victims of physical and sexual abuse have difficulties seeking and retaining gainful employment, trusting others, developing intimate relationships, and regulating their anger and behavior [14]. The higher number of convictions in patients with methamphetamine dependence suggests that methamphetamine dependence is complicated by antisocial personality disorder.

With regard to family histories of alcohol dependence, drug dependence, and psychiatric disorders, patients with alcohol dependence had higher ratios of having a father, paternal uncle, and brother with alcohol-related problems. Patients with methamphetamine dependence had higher ratios of having a brother with drug-related problems. These significant results were found only with male relatives, and substance (alcohol or drug) use that became a problem for patients was common when substances were used by their male relatives. However, because these results may have been affected by the high prevalence of individuals with alcohol or drug dependence in the male population [15], these results should be interpreted with caution. Additionally, information about antisocial characteristics among not only the patients but also their families may be worth collecting in future studies to ascertain differences in the interactions between parents and children with substance dependence.

Based on the above results from patients with alcohol dependence, unestablished family relationships over time influenced a wide range of problems, especially the severity of psychiatric problems. This result suggests the usefulness of psychological therapy for treating family dysfunction and self-help group therapy. In patients with methamphetamine dependence, unestablished relationships with their father over the years may not have been linked to their present severity of substance dependence in ASI CSs. Moreover, not simply relationships with specific family members but overall family/social relationships may be related to severity in ASI CSs (e.g., Psychiatric, Medical, and Legal problems). Given the result that patients with methamphetamine dependence often lived with their parents, investigating the effect of bad relationships with their father on relationships with their brother with drug-related problems may be important. Furthermore, verifying the possibility that patients with methamphetamine dependence may not often establish good relationships with their father because of their experiences of abuse by their parents may be meaningful in future studies.

A previous study suggested the importance of distinguishing between alcohol and drug dependence disorders and examining their differential etiological pathways [16]. The present study may also suggest the necessity of separately investigating the association between family relationships and various problems related to substance dependence in alcohol dependence and methamphetamine dependence. The results of the present study may provide support for the possibility that the results of the ASI as an intake instrument may be an indicator of early intervention for family and social problems, and personalized programs that augment usual interventions may be useful.

Although this study provided useful new insights, it has a few limitations. First, the sample did not contain female patients. Role differences in a family may exist between males and females. Future studies should assess female patients. Second, the uniformity of the participants in this study may be problematic, including differences in age and present status (*i.e.*, inpatient, outpatient, or recovering individual) between the alcohol dependence group and methamphetamine dependence group. Third, this study utilized a cross-sectional design, so we could not establish a causal relationship between family relationships and problems related to alcohol or drug dependence. However, the results of this study may be beneficial for future longitudinal studies.

## Figures and Tables

**Table 1:** Substances that participants with drug dependence in this study mainly used.

Drug	%
Methamphetamine	85.00
Cannabis	6.25
Inhalants	3.75
Analgesics/hypnotics/tranquilizers	2.50
Antitussive drugs	1.25
Hallucinogens (e.g., lysergic acid diethylamide)	1.25

**Table 2:** Participant characteristics.

Characteristic	Alcohol dependence (*n* = 321)	Methamphetamine dependence (*n* = 68)	*p*
Mean age (years [SD])	49.46 (10.34)	34.82 (8.51)	< 0.0001 *
*Education*			
Education (mean years [SD])	11.75 (2.69)	11.87 (2.32)	n.s.
% Junior high school graduate	29.91	14.71	< 0.05 *
% Some high school	9.66	25.00	< 0.05 *
% High school graduate	31.15	30.88	n.s.
% Some college	7.48	11.76	n.s.
% College graduate	18.38	13.24	n.s.
% Unclear	3.43	4.41	n.s.
*Employment (past 3 years)*			
% Full-time	69.47	41.18	< 0.05 *
% Part-time	10.59	25.00	< 0.05 *
% Retired	6.85	0.00	< 0.05 *
% Unemployed	10.90	25.00	< 0.05 *
% Other	2.18	8.82	< 0.05 *
% Public assistance recipient (past 30 days)	8.41	16.18	n.s.
*Marital status*			
% Married	54.21	8.82	< 0.05 *
% Never married	21.18	66.18	< 0.05 *
% Separated/Widowed/Divorced	24.61	25.00	n.s.
*Cohabitation*			
% With family	46.11	10.29	< 0.05 *
% With spouse	14.64	10.29	n.s.
% With parents	13.40	39.71	< 0.05 *
% Alone	21.81	19.12	n.s.
% Other	4.05	20.59	< 0.05 *
*Years of current cohabitation*			
% < 10 years	41.32	65.57	< 0.05 *
% 10–20 years	22.08	11.48	< 0.05 *
% > 20 years	36.59	22.95	< 0.05 *
*Abuse*			
% Emotional abuse	22.19	14.93	0.25
% Physical abuse	6.85	17.91	0.007 *
% Sexual abuse	0.00	0.00	-
*Psychiatric symptoms*			
% Serious depression	15.26	14.71	1.00
% Serious anxiety or tension	24.61	32.35	0.22
% Hallucinations	2.80	14.71	0.0003 *
% Trouble understanding, concentrating, or remembering	12.46	32.35	0.0002 *
% Trouble controlling violent behavior	4.05	10.29	0.06
% Serious thoughts of suicide	18.07	16.18	0.86
% Attempted suicide	3.12	4.40	0.71
Number of convictions (mean years [SD])	0.14 (0.74)	1.32 (1.79)	< 0.0001 *

SD, standard deviation;

* significant difference.

**Table 3:** Correlations between ASI composite scores after controlling for age.

*Alcohol dependence*		Employment	Alcohol use	Drug use	Legal	Family/Social	Psychiatric
Medical	*r*	0.08	−0.13	0.08	0.05	0.03	0
	*p*	0.2	0.03	0.16	0.38	0.62	0.97

Employment	*r*		0.01	0.07	0.13	0.12	−0.02
	*p*		0.85	0.26	0.02	0.04	0.69

Alcohol use	*r*			−0.04	0.01	0.14	0.09
	*p*			0.51	0.85	0.02	0.14

Drug use	*r*				0	0.14	0.26
	*p*				0.96	0.02	< 0.0001 ^*^

Legal	*r*					0.02	−0.07
	*p*					0.69	0.23

Family/Social	*r*						0.28
	*p*						< 0.0001 ^*^

*Methamphetamine dependence*		Employment	Alcohol use	Drug use	Legal	Family/Social	Psychiatric
Medical	*r*	0.17	0.04	−0.04	0.19	0.33	0.33
	*p*	0.17	0.76	0.78	0.12	0.007 *	0.008 *

Employment	*r*		−0.04	0.22	−0.01	0.05	0.40
	*p*		0.76	0.07	0.92	0.71	0.001 *

Alcohol use	*r*			0.12	−0.06	0.20	0.06
	*p*			0.34	0.61	0.11	0.65

Drug use	*r*				−0.05	0.07	0.40
	*p*				0.68	0.60	0.0009 *

Legal	*r*					0.35	0.09
	*p*					0.004 *	0.48

Family/Social	*r*						0.43
	*p*						0.0004 *

* Significant correlation (*p* < 0.01).

**Table 4:** Comparison of ratios of each psychiatric symptom between groups of high and low CSs of Family/Social relationship problems.

*Alcohol dependence*	Family/Social		

	High	Low	*p*
Serious depression (%)	22.62	9.41	0.001 ^*^
Serious anxiety or tension (%)	32.56	19.08	0.02 ^*^
Hallucinations (%)	2.33	3.82	0.72
Trouble understanding, concentrating, or remembering (%)	14.73	12.21	0.59
Trouble controlling violent behavior (%)	5.43	3.88	0.77
Serious thoughts of suicide (%)	25.78	12.98	0.01 ^*^
Attempted suicide (%)	3.10	3.08	1.00

*Methamphetamine dependence*	Family/Social		

	High	Low	*p*
Serious depression (%)	21.88	6.06	0.08
Serious anxiety or tension (%)	39.39	24.24	0.29
Hallucinations (%)	18.18	12.12	0.73
Trouble understanding, concentrating, or remembering (%)	42.42	24.24	0.19
Trouble controlling violent behavior (%)	18.18	3.03	0.10
Serious thoughts of suicide (%)	18.18	12.12	0.73
Attempted suicide (%)	3.03	3.03	1.00

* Significant difference.

**Table 5:** Family history of alcohol dependence, methamphetamine dependence, and psychiatric disorders.

Relation	Alcohol dependence	Methamphetamine dependence	*p*
*Grandmother (maternal) (%)*			
Alcohol	1.44	2.04	0.57
Drug	0.00	0.00	—
Psychiatric disorder	0.00	0.00	—
*Grandfather (maternal) (%)*			
Alcohol	15.42	6.12	0.11
Drug	0.00	0.00	—
Psychiatric disorder	0.00	0.00	—
*Mother (%)*			
Alcohol	1.30	1.92	0.54
Drug	0.33	0.00	1.00
Psychiatric disorder	2.64	7.41	0.09
*Aunt (maternal) (%)*			
Alcohol	2.83	2.04	1.00
Drug	0.41	2.08	0.30
Psychiatric disorder	0.41	2.04	0.31
*Uncle (maternal) (%)*			
Alcohol	14.45	6.00	0.17
Drug	0.79	0.00	1.00
Psychiatric disorder	1.19	3.92	0.20
*Sisters (%)*			
Alcohol	3.73	2.44	1.00
Drug	0.00	2.50	0.14
Psychiatric disorder	2.93	2.50	1.00
*Grandmother (paternal) (%)*			
Alcohol	1.47	0.00	1.00
Drug	0.00	1.96	0.20
Psychiatric disorder	0.98	3.85	0.18
*Grandfather (paternal) (%)*			
Alcohol	18.37	7.84	0.09
Drug	0.00	1.96	0.20
Psychiatric disorder	0.50	1.96	0.37
*Father (%)*			
Alcohol	36.33	16.67	0.005 *
Drug	1.02	0.00	1.00
Psychiatric disorder	1.72	0.00	1.00
*Aunt (paternal) (%)*			
Alcohol	3.45	0.00	0.36
Drug	0.00	0.00	-
Psychiatric disorder	0.87	2.08	0.44
*Uncle (paternal) (%)*			
Alcohol	20.87	5.88	0.009 *
Drug	0.00	1.96	0.18
Psychiatric disorder	0.44	0.00	1.00
*Brothers (%)*			
Alcohol	25.94	4.88	0.002 *
Drug	0.00	9.52	0.0005 *
Psychiatric disorder	1.68	4.88	0.22

* Significant difference.

**Table 6:** Comparisons of the ratios of good family relationships between patients with alcohol dependence and patients with methamphetamine dependence.

	Alcohol dependence	Methamphetamine dependence	*p*
Mother (%)	76.07	64.71	0.07
Father (%)	70.55	43.08	< 0.0001 *
Brothers/sisters (%)	72.43	60.66	0.09
Partner (%)	62.89	58.00	0.52
Children (%)	72.29	53.33	0.14
Friends (%)	77.74	69.49	0.18

* Significant difference.

**Table 7:** Comparison of severity of addiction between good and bad family relationships in patients with alcohol dependence.

	Mother			Father		
		
	Good relationship	Bad relationship	*p*	Good relationship	Bad relationship	*p*
Medical	0.22 (0.29)	0.29 (0.33)	0.12	0.22 (0.28)	0.29 (0.34)	0.14
Employment	0.53 (0.28)	0.55 (0.29)	0.64	0.53 (0.29)	0.55 (0.27)	0.60
Alcohol use	0.55 (0.22)	0.54 (0.22)	0.63	0.55 (0.23)	0.55 (0.23)	0.78
Drug use	0.01 (0.04)	0.01 (0.03)	0.80	0.01 (0.04)	0.01 (0.03)	0.66
Legal	0.004 (0.03)	0.01 (0.05)	0.42	0.004 (0.03)	0.004 (0.04)	0.99
Family/Social	0.23 (0.22)	0.25 (0.21)	0.57	0.23 (0.22)	0.23 (0.20)	0.88
Psychiatric	0.13 (0.18)	0.20 (0.24)	0.01 ^*^	0.12 (0.18)	0.20 (0.23)	0.01 ^*^

	Brothers and Sisters			Partner		
		
	Good relationship	Bad relationship	*p*	Good relationship	Bad relationship	*p*
Medical	0.22 (0.29)	0.26 (0.32)	0.30	0.26 (0.31)	0.27 (0.31)	0.77
Employment	0.51 (0.27)	0.61 (0.30)	0.00 ^*^	0.48 (0.29)	0.60 (0.27)	0.00 ^*^
Alcohol use	0.53 (0.22)	0.57 (0.23)	0.18	0.55 (0.22)	0.53 (0.22)	0.46
Drug use	0.01 (0.04)	0.01 (0.04)	0.60	0.01 (0.04)	0.01 (0.04)	0.26
Legal	0.003 (0.03)	0.01 (0.05)	0.32	0.005 (0.04)	0.01 (0.04)	0.67
Family/Social	0.22 (0.22)	0.26 (0.19)	0.17	0.19 (0.19)	0.33 (0.23)	0.00 ^*^
Psychiatric	0.13 (0.17)	0.20 (0.25)	0.02 ^*^	0.13 (0.18)	0.17 (0.22)	0.09

	Children			Friends		
		
	Good relationship	Bad relationship	*p*	Good relationship	Bad relationship	*p*
Medical	0.25 (0.31)	0.32 (0.32)	0.11	0.24 (0.30)	0.31 (0.34)	0.15
Employment	0.50 (0.30)	0.56 (0.27)	0.13	0.51 (0.29)	0.59 (0.29)	0.07
Alcohol use	0.54 (0.23)	0.55 (0.21)	0.81	0.55 (0.22)	0.55 (0.22)	1.00
Drug use	0.01 (0.04)	0.004 (0.02)	0.38	0.01 (0.04)	0.01 (0.02)	0.43
Legal	0.003 (0.02)	0.01 (0.04)	0.52	0.004 (0.03)	0.01 (0.05)	0.36
Family/Social	0.22 (0.22)	0.27 (0.19)	0.14	0.23 (0.21)	0.23 (0.22)	0.82
Psychiatric	0.13 (0.19)	0.15 (0.21)	0.70	0.13 (0.18)	0.22 (0.26)	0.02 *

* Significant difference.

**Table 8:** Comparison of severity of addiction between good and bad family relationships in patients with methamphetamine dependence.

	Mother			Father		
		
	Good relationship	Bad relationship	*p*	Good relationship	Bad relationship	*p*
Medical	0.05 (0.17)	0.13 (0.24)	0.21	0.04 (0.13)	0.12 (0.25)	0.08
Employment	0.67 (0.22)	0.74 (0.25)	0.28	0.70 (0.21)	0.70 (0.25)	0.99
Alcohol use	0.12 (0.19)	0.19 (0.27)	0.26	0.10 (0.20)	0.16 (0.23)	0.27
Drug use	0.09 (0.09)	0.12 (0.12)	0.27	0.08 (0.09)	0.12 (0.11)	0.19
Legal	0.02 (0.07)	0.04 (0.13)	0.29	0.01 (0.06)	0.03 (0.11)	0.32
Family/Social	0.14 (0.13)	0.23 (0.20)	0.05	0.13 (0.13)	0.21 (0.18)	0.06
Psychiatric	0.23 (0.24)	0.32 (0.27)	0.17	0.24 (0.23)	0.30 (0.27)	0.34

	Brothers/Sisters			Partner		
		
	Good relationship	Bad relationship	*p*	Good relationship	Bad relationship	*p*
Medical	0.05 (0.14)	0.12 (0.24)	0.18	0.08 (0.20)	0.05 (0.14)	0.54
Employment	0.66 (0.21)	0.77 (0.25)	0.08	0.62 (0.26)	0.73 (0.21)	0.10
Alcohol use	0.10 (0.19)	0.21 (0.26)	0.08	0.19 (0.24)	0.13 (0.24)	0.43
Drug use	0.09 (0.09)	0.12 (0.12)	0.31	0.09 (0.12)	0.09 (0.09)	0.93
Legal	0.02 (0.08)	0.04 (0.11)	0.51	0.04 (0.11)	0.002 (0.01)	0.09
Family/Social	0.15 (0.16)	0.23 (0.17)	0.07	0.18 (0.14)	0.17 (0.14)	0.80
Psychiatric	0.23 (0.26)	0.30 (0.24)	0.36	0.24 (0.26)	0.24 (0.23)	0.94

	Children			Friends		
		
	Good relationship	Bad relationship	*p*	Good relationship	Bad relationship	*p*
Medical	0.11 (0.20)	0.07 (0.19)	0.71	0.08 (0.21)	0.09 (0.20)	0.77
Employment	0.54 (0.34)	0.66 (0.27)	0.42	0.69 (0.24)	0.69 (0.23)	0.94
Alcohol use	0.16 (0.18)	0.34 (0.34)	0.24	0.13 (0.21)	0.17 (0.25)	0.55
Drug use	0.04 (0.06)	0.07 (0.11)	0.52	0.09 (0.10)	0.12 (0.12)	0.46
Legal	0.03 (0.09)	0.00 (0.00)	0.42	0.02 (0.09)	0.04 (0.11)	0.62
Family/Social	0.15 (0.13)	0.15 (0.12)	0.89	0.18 (0.15)	0.16 (0.16)	0.69
Psychiatric	0.14 (0.19)	0.14 (0.19)	0.97	0.28 (0.24)	0.69	

* Significant difference.
